# Supplementary material for: Radioimmune Imaging of α4β7 Integrin and TNFα for Diagnostic and Therapeutic Applications in Inflammatory Bowel Disease
Source: Pharmaceutics. 2023 Mar 2;15(3):817. doi: 10.3390/pharmaceutics15030817 (PMC10051745; doi:10.3390/pharmaceutics15030817)

pharmaceutics MDPI

Supplementary data

Table S1: Biodistribution of radiolabelled mAbs in mice. Data are shown as mean %ID/g±SD.

	Anti-TNFα (CTRL mice)				Anti-integrin (CTRL mice)				Control isotype (CTRL mice)			
	6 h		24 h		6 h		24 h		6 h		24 h	
	%ID/g	SD	%ID/g	SD	%ID/g	SD	%ID/g	SD	%ID/g	SD	%ID/g	SD
Blood	9.15	1.10	2.89	1.02	62.86	10.45	27.89	6.54	30.22	3.59	18.85	1.28
Colon (ATDR)	1.58	0.35	0.97	0.38	10.41	4.62	12.67	4.90	3.33	0.69	3.68	0.30
Small Bowel	2.03	0.14	1.52	1.41	32.23	1.79	23.44	8.89	5.34	0.26	4.76	0.56
Kidneys	8.83	2.36	7.50	4.50	27.98	3.10	16.50	0.14	16.68	2.71	13.27	0.91
Spleen	24.54	9.02	17.09	5.82	267.41	39.83	172.11	83.22	22.14	2.04	19.91	2.88
Stomach	4.03	0.48	2.67	1.37	8.72	2.46	4.16	0.72	5.98	1.79	3.13	0.80
Liver	34.97	6.84	21.92	9.36	47.16	5.44	41.76	31.65	22.72	1.66	20.34	1.20
Muscle	0.75	0.33	0.27	0.10	3.35	1.14	2.69	0.81	1.43	0.02	2.00	0.18
Bone	4.12	1.09	2.43	0.81	40.75	10.97	21.69	8.92	4.73	0.57	4.65	1.07
Lungs	3.76	0.33	1.41	0.30	36.59	3.24	16.25	1.26	11.63	2.98	9.05	1.88
Heart	2.47	0.32	0.93	0.30	14.86	1.80	7.64	1.44	8.77	0.64	6.79	0.97

	Anti-TNFα (DSS mice)				Anti-integrin (DSS mice)				Control isotype (DSS mice)			
	6 h		24 h		6 h		24 h		6 h		24 h	
	%ID/g	SD	%ID/g	SD	%ID/g	SD	%ID/g	SD	%ID/g	SD	%ID/g	SD
Blood	9.64	0.19	2.97	0.36	61.51	11.92	25.13	14.95	32.19	5.21	13.55	9.83
Colon (ATDR)	2.39	0.66	1.55	0.88	18.32	13.06	22.41	8.91	5.62	1.14	10.97	4.06
Small Bowel	2.23	0.42	0.89	0.05	19.44	8.79	21.04	5.75	4.71	0.47	4.24	3.99
Kidneys	6.15	4.86	5.10	0.05	48.93	20.41	20.94	3.99	18.15	2.97	9.36	7.77
Spleen	29.26	1.01	18.50	5.30	76.13	81.43	77.22	8.48	27.46	7.23	13.80	10.64
Stomach	6.15	1.12	3.52	2.44	12.81	8.32	11.51	7.74	7.16	3.55	8.68	2.41
Liver	40.35	2.08	26.37	3.99	43.20	10.56	40.32	4.66	24.42	3.86	15.25	13.17
Muscle	0.90	0.02	0.26	0.07	3.34	0.80	2.39	0.13	1.67	0.19	0.63	0.55
Bone	3.54	0.19	1.94	0.57	32.76	16.54	32.41	4.38	6.41	3.38	3.08	2.15
Lungs	3.72	0.37	1.38	0.07	53.53	33.09	22.57	3.88	11.04	1.04	7.09	1.10
Heart	2.87	0.46	0.86	0.06	23.05	15.93	9.07	2.07	8.90	4.46	4.61	3.80

%ID/g = percentage of Injected Dose per gram of tissue; SD = Standard Deviation; CTRL = Control; DSS = Dextran Sulfate Sodium; ATDR = Ascending, Transvers, Descending, Recto-sigmoidal colon.

Figure S1: Left: correlation between immunohistochemistry staining for $\alpha_4\beta_7$ integrin (mean score of 5 rings from each block) and radioactivity counted in each block (expressed as %ID) of a DSS-mouse injected with ^{99m}Tc-anti-$\alpha_4\beta_7$ integrin mAb, 24h before. A correlation was found between the two parameters highlighting the specificity of ^{99m}Tc-anti-$\alpha_4\beta_7$ integrin mAb to $\alpha_4\beta_7$ integrin in tissue. Right: the figure shows the large bowel with superimposed the gamma-camera image of it. Ascending colon, transvers colon, descending colon, sigma-rectum, were each cut in 4 blocks and counted for radioactivity. Then, each block was cut in 5 rings and stained for immunohistochemistry. Black values correspond to counts in each block. Values in green represents the mean score of $\alpha_4\beta_7$ integrin expression in 5 rings of the same block.

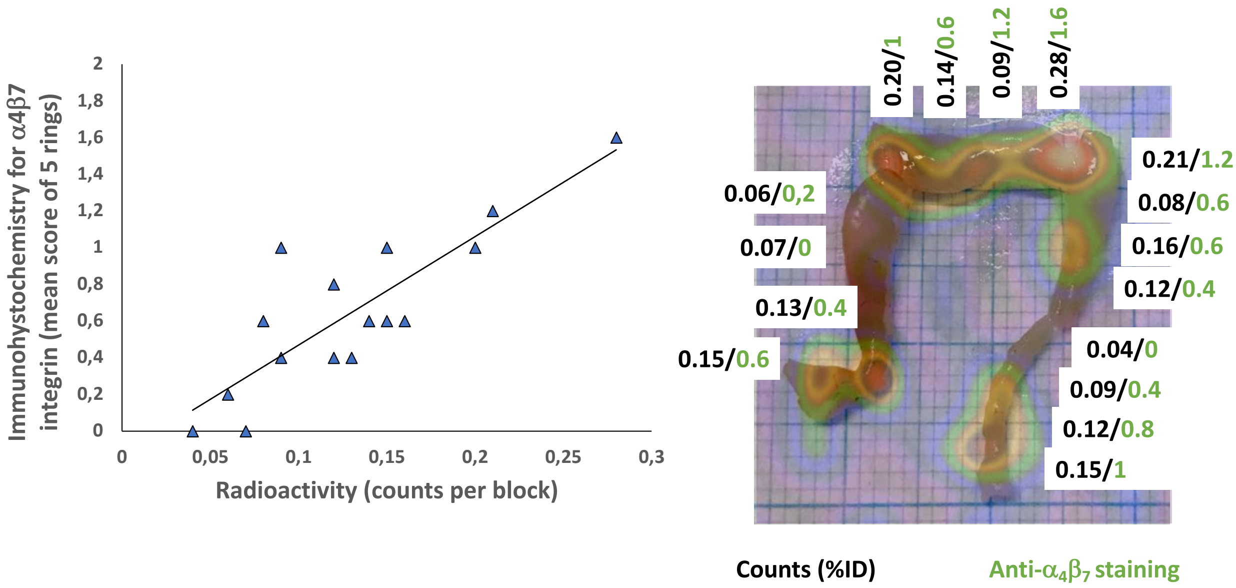

Figure S2: Left: correlation between immunohistochemistry staining for TNFα (mean score of 5 rings from each block) and radioactivity counted in each block (expressed as %ID) of a DSS-mouse injected with ^{99m}Tc-anti-TNFα mAb, 24h before. A correlation was found between the two parameters highlighting the specificity of ^{99m}Tc-anti-TNFα mAb to TNFα in tissue. Right: the figure shows the large bowel with superimposed the gamma-camera image of it. Ascending colon, transvers colon, descending colon, sigma-rectum, were each cut in 4 blocks and counted for radioactivity. Then, each block was cut in 5 rings and stained for immunohistochemistry. Black values correspond to counts in each block. Values in red represents the mean score of TNFα expression in 5 rings of the same block.

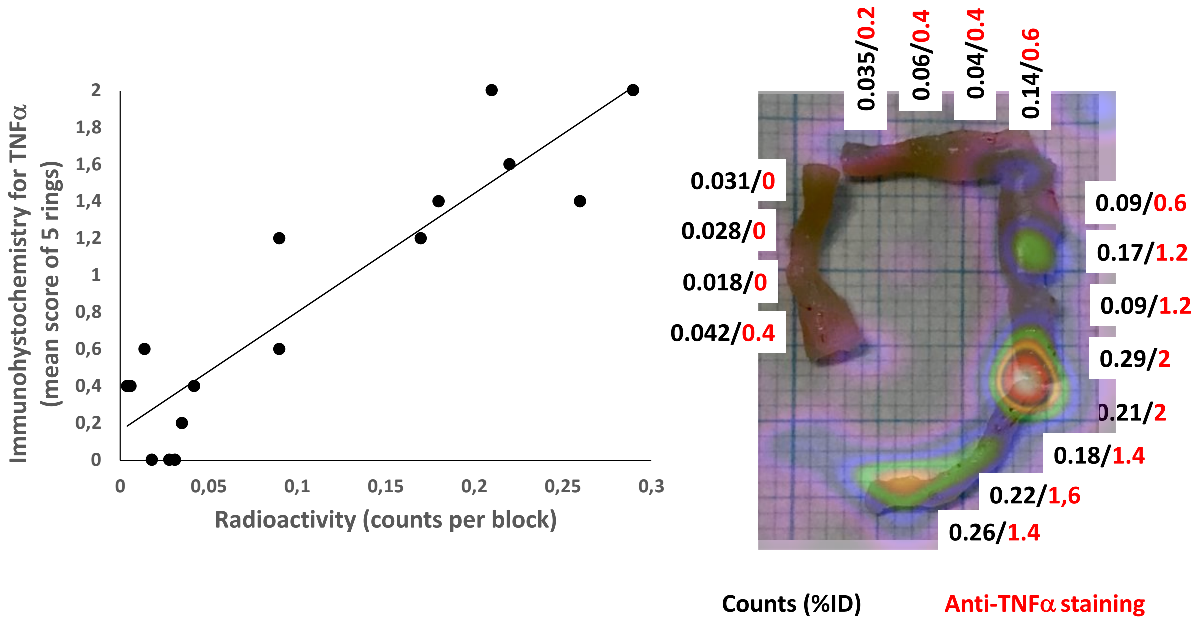

Figure S3: Left: correlation between immunohistochemistry staining for $\alpha_4\beta_7$ integrin (circles) and for TNFα (triangles), (mean score of 5 rings from each block) and radioactivity counted in each block (expressed as %ID) of a DSS-mouse injected with ^{99m}Tc-anti-IgG$_{2a}$ mAb, 24h before. No correlation was found between the two parameters (dotted lines) highlighting that the accumulation of ^{99m}Tc-anti-IgG$_{2a}$ mAb is not related to presence of $\alpha_4\beta_7$ integrin or TNFα in tissue. Right: the figure shows the large bowel with superimposed the gamma-camera image of it. Ascending colon, transvers colon, descending colon, sigma-rectum, were each cut in 4 blocks and counted for radioactivity. Then, each block was cut in 5 rings and stained for immunohistochemistry. Black values correspond to counts in each block. Values in green represents the mean score of $\alpha_4\beta_7$ integrin expression in 5 rings of the sand values in red represents the mean score of TNFα expression.

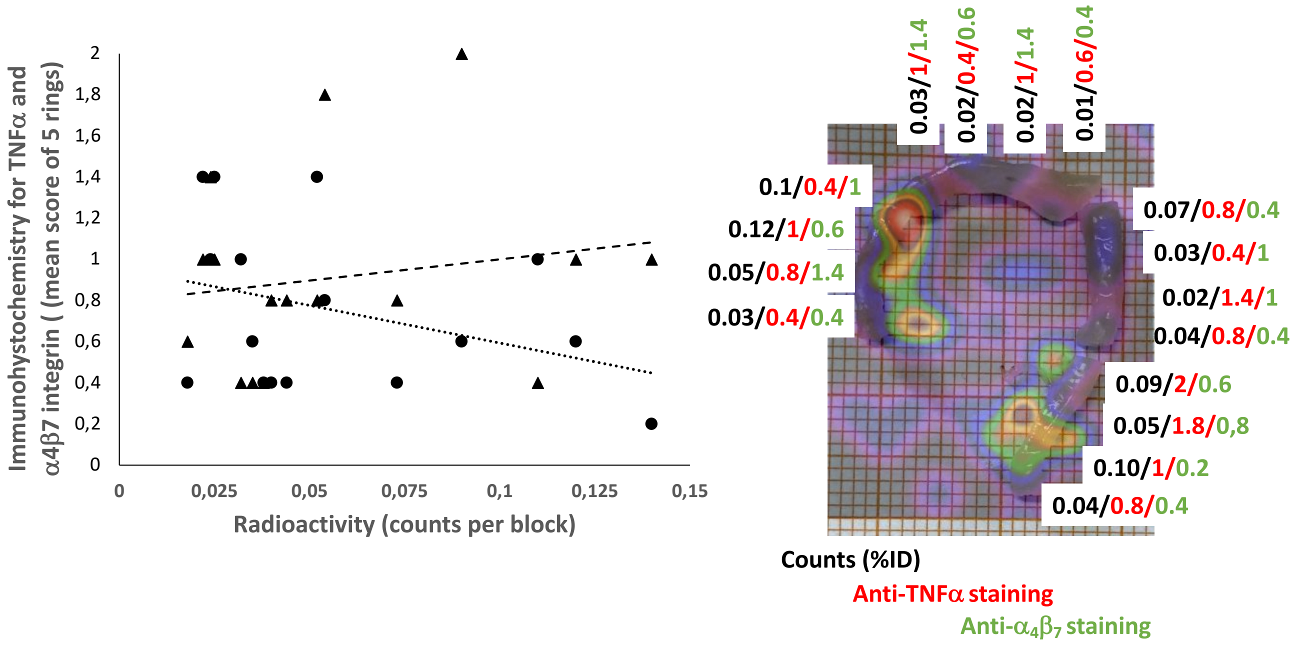

Supplement: Supplementary file 1 [file pharmaceutics-15-00817-s001.zip › pharmaceutics-2193241-supplementary.pdf]
